# Taste and smell function in Wolfram syndrome

**DOI:** 10.1186/s13023-020-1335-7

**Published:** 2020-02-22

**Authors:** Raul Alfaro, Tasha Doty, Anagha Narayanan, Heather Lugar, Tamara Hershey, M. Yanina Pepino

**Affiliations:** 1 Department of Food Science and Human Nutrition, University of Illinois at Urbana Champaign, Urbana, IL USA; 2 Department of Psychiatry, School of Medicine, Washington University, St. Louis, MO USA; 3 Department of Radiology, School of Medicine, Washington University, St. Louis, MO USA; 4 Division of Nutritional Sciences, University of Illinois at Urbana Champaign, Urbana, IL USA

**Keywords:** Wolfram syndrome, DIDMOAD, UPSIT, Sniffin’ sticks, Olfaction, Taste, Neurodegeneration

## Abstract

**Background:**

Wolfram syndrome is a rare genetic disease characterized by insulin-dependent diabetes, optic nerve atrophy, sensorineural hearing loss and neurodegeneration. Although olfactory dysfunction, a classical clinical marker of neurodegenerative processes, has been reported in Wolfram syndrome, its use as a clinical marker in Wolfram is limited due to data scarcity. In addition, it is unknown whether Wolfram syndrome affects the sense of taste.

**Methods:**

Smell and taste perception were assessed in participants with Wolfram syndrome (*n* = 40) who were 15.1 ± 6.0 years of age (range: 5.1–28.7 years) and two sex- and age-matched control groups: one group with type 1 diabetes mellitus (T1D; *n* = 25) and a healthy control group (HC; *n* = 29). Smell sensitivity was assessed by measuring n-butanol detection thresholds and smell identification by using the University of Pennsylvania Smell Identification Test (UPSIT). Taste function was assessed using NIH Toolbox, which includes the assessment of sucrose (sweet) taste preference, and perceived intensity of sucrose, sodium chloride (salty), and quinine hydrochloride (bitter) both in the tip of the tongue (regional test) and the whole mouth.

**Results:**

Smell sensitivity was not significantly different among groups; however, smell identification was impaired in Wolfram syndrome, as reflected by significantly lower UPSIT scores in Wolfram syndrome compared to HC and T1D (*P* < 0.001). Compared to participants in the control groups, participants with Wolfram syndrome had a blunted perception of sweetness and saltiness when taste stimuli were applied regionally (*P* < 0.05), but differences in perceived intensity were no longer significant among groups when taste stimuli were tasted with the whole mouth. Groups preferred similar sucrose concentrations.

**Conclusion:**

Wolfram syndrome was associated with olfactory dysfunction. However, the olfactory dysfunction was qualitative (related to smell identification) and not secondary to olfactory insensitivity or diabetes, suggesting is arising from dysfunction in central olfactory brain regions. In contrast to olfaction, and despite decreased perception of taste intensity in the anterior tongue, the sense of taste was overall well-conserved in individuals with Wolfram syndrome. Future longitudinal studies of taste and smell perception in Wolfram syndrome will be important to determine the use of the chemical senses as clinical markers of disease progression.

## Background

Wolfram syndrome is a rare genetic disease with an estimated prevalence of 1 in 770,000 [1] caused by mutations in the genes *WFS1* [2] or, less commonly, *WFS2* [3]. These mutations disrupt normal endoplasmic reticulum functioning [4] causing cellular apoptosis in different body tissues [5]. Hallmarks of Wolfram syndrome include insulin dependent diabetes [6], diabetes insipidus [7], loss of visual acuity due to optic nerve atrophy, sensorineural hearing loss [8] and other neurological complications [1, 9].

While neurological complications in Wolfram syndrome were traditionally thought to be the result of neurodegenerative processes, appearing at more advanced stages of disease, recent data suggest that some brain abnormalities, including decreased brainstem volume, arise early in the disease, some prior to the development of significant clinical symptoms [10]. Therefore, Wolfram syndrome presents features of both altered neurodevelopment and neurodegenerative processes [11].

One important clinical marker and one of the earliest signs of common neurodegenerative diseases such as Alzheimer [12, 13] and Parkinson disease [14, 15] is olfactory dysfunction. Although olfactory dysfunction has been associated with Wolfram syndrome in clinical reports [16–19] data from standardized tests measuring sense of smell is very limited. Identification of common smells was assessed in 19 Wolfram syndrome participants from the Wolfram Syndrome Research Clinic in Washington University at St. Louis, MO [20, 21]. Using the University of Pennsylvania Smell Identification Test (UPSIT), we previously reported that 72% of patients with Wolfram syndrome had olfactory defects [20]. However, because UPSIT measures only how well an individual can identify odorants, it is unknown whether the Wolfram syndrome group’s UPSIT performance is secondary to impairments in the peripheral nervous system (smell sensitivity), solely due to impairment at the central level (smell identification), or a combination of both [22].

Wolfram syndrome is known to affect most senses (vision, hearing, smell). However, we do not know if taste is affected, independent of olfaction. The first neural relay for the central gustatory pathway is in the brainstem, a region severely affected in Wolfram syndrome, thus it is plausible that taste could be affected. Remarkably, unlike the other senses, which carry information to the brain by one single cranial nerve, taste sensations are conveyed by three cranial nerves (facial, glossopharyngeal and vagal). The facial nerve innervates the anterior two-thirds of the tongue; however, a blunted or altered taste sensation mediated by this nerve may be unnoticed when evaluating taste perception in the whole mouth due to central release of inhibition from other oral sensory cranial nerves. The regional application of taste at the tip of the tongue isolates taste perception that is carried by the facial nerve and therefore, the comparison of taste perception in the whole mouth versus that in the tip of the tongue allows detection of specific nerve damage. Defining the nature of taste and smell deficits in Wolfram syndrome would expand our understanding of the specific or global nature of sensory deficits in this complex disorder and could provide biomarkers to evaluate disease progression as well as effectiveness of potential treatments.

The primary goals of the present study were to test the hypotheses that Wolfram syndrome was associated with smell and taste dysfunction. Accordingly, we evaluated smell and taste function using validated psychophysical tests that included regional and whole mouth procedures for taste perception, as well as measures of both smell sensitivity and smell identification in patients with Wolfram syndrome and in two age and sex equivalent healthy (HC) and type 1 diabetes (T1D) control groups.

## Methods

### Participants and study design

Participants with genetically confirmed diagnosis of Wolfram syndrome were recruited via self or physician referral to attend the annual Washington University Wolfram syndrome Research Clinic. In addition, two control groups were recruited: a healthy control group (HC) and a type 1 diabetes group (T1D). Participants with Wolfram syndrome attended the Wolfram Research Clinic between 2010 and 2017 and participants in the control groups were recruited throughout the years 2013 and 2016. Several participants from the three study groups completed testing procedures in consecutive years, but only data from each participant’s initial evaluation of both UPSIT and Sniffin Stick was included in this cross-sectional sample. The three study groups (Wolfram, HC, and T1D) underwent the same psychophysical assessments of smell and taste, described in detail below. In addition to completing taste and smell assessments, participants with Wolfram syndrome were evaluated with a comprehensive battery of test across multiple domains. The Human Research Protection Office at Washington University in St. Louis approved the study protocol. Participants under 18 years of age gave informed assent and their parents or legal guardians gave informed written consent, and those who were 18 years old or older gave informed written consent.

### Smell function

#### Olfactory sensitivity

Olfactory detection threshold measures the lowest concentration of a smell that can be detected. Olfactory detection thresholds were measured for n-butanol (CAS# 71–36-3) using previously described and validated methods [23]. This procedure involved exposing participants to randomized series of three felt-tip pens (“Sniffin’ Sticks”) in which one had a determined concentration of n-butanol dissolved in deionized water and the other two had deionized water only (i.e. blanks). Participants were blindfolded to prevent visual identification of the blank sticks. On each trial, the uncapped pen tip was exposed for approximately 3 s at two centimeters from the participant’s nose. The participant was instructed to sniff in and determine which of the sticks had an odor. Presentation of the triplets occurred approximately every 30 s. Overall, there were 16 n-butanol concentrations (16 successive dilutions with a 1:2 ratio; highest concentration was 4% v/v). A simple staircase method was used so that the concentration in the odor containing stick increased after an incorrect answer and decreased after the participant correctly discerned the stick with the odorant in two consecutive trials. The points at which the concentration sequence changed directions are considered “reversals” of the staircase. Testing continued until the participant achieved seven reversals and the mean of the last four reversals was then calculated as the n-butanol threshold. Based on previous data [24] an n-butanol threshold dilution level below 10% for their age and sex was considered indicative of hyposmia or abnormal smell functionality.

#### Olfactory identification

Participants completed the 40-item UPSIT [25]. The test contains four booklets, each with 10 pages containing a “scratch and sniff” box with microencapsulated odorants. The task for the participants was to scratch the box and then indicate which of four response alternatives written on the page best matched the perceived smell. When participants had vision limitations, the investigator read in a loud voice the options to the participant immediately after he/she smelled the scratch and sniff box. Standardized UPSIT scores from normative data, adjusted for age and sex, were used to consider a score under the 10th percentile to be indicative of hyposmia or olfactory dysfunction [26]. To control for cultural differences affecting recognition or exposure to certain odors, we also calculated scores using a subset of 12 odors that are commonly found in every culture around the world and that are validated as a “Cross-Cultural Smell Identification Test (CC-SIT)” [27]. Data on UPSIT on a subset of subjects included in this manuscript (19 WFS, 24 HC and 25 T1D) has been published [20, 21].

### Taste function

#### Taste quality and intensity

We assessed both taste quality and intensity of varying concentrations of sucrose, sodium chloride and quinine hydrochloride using a regional (tip of the tongue) and a whole mouth taste presentation method, as recommended in the NIH Toolbox for Assessment of Neurological and Behavioral Function [28]. Participants rated the strength of their taste perception on a generalized labeled magnitude scale (gLMS). The gLMS is a measure of perceived intensity with seven anchor labels provided (Strongest of any kind, Very strong, Strong, Moderate, Weak, Barely detectable, No sensation) [29, 30]. Before using the gLMS scale to assess the taste of solutions, subjects were trained on the use of the scale and asked to rank light intensities in practice trials (i.e. the intensity of light in a candle-lit restaurant, in a well-lit room and of the strongest/brightest light they have ever seen) [28]. Data on taste intensity includes only participants who were at least 12 years of age at time of testing because the gLMS scale has not been validated for younger ages (Table 1).

**Table 1:** Age, sex and number of participants in the Wolfram syndrome group and control groups for each of the completed assessments

	HC		T1D		Wolfram	
	Age (years ± SD)	n (male/female)	Age (years ± SD)	n (male/female)	Age (years ± SD)	n (male/female)
Sucrose Preference	14.4 ± 5.3	15/11	13.8 ± 4.8	9/13	14.3 ± 5.8	16/23
Taste Intensity	17.1 ± 3.7	11/7	16.6 ± 4.3	6/7	17.5 ± 4.8	9/19
UPSIT	14.8 ± 5.3	15/14	14.4 ± 4.7	10/15	15.1 ± 6.0	17/23
Sniffin’ Sticks	13.9 ± 4.9	8/10	15.1 ± 5.1	7/11	15.2 ± 6.0	16/23

*HC* Healthy control, *T1D* Type 1 Diabetes, *SD* Standard deviation, *UPSIT* University of Pennsylvania Smell Identification Test

We first assessed regional taste function by applying the taste stimulus in a cotton swab soaked with the taste solution in a semicircular motion around the tip of the tongue. Sucrose (90 mm, 350 mm, and 1050 mm), sodium chloride (NaCl) (100 mm, 320 mm, and 1000 mm), and quinine hydrochloride (0.01 mm, 0.03 mm, and 1 mm) were used as the sweet, salty, and bitter stimulus respectively. The order of presentation of solutions was randomized with exception of the highest quinine concentration, which was always presented at the end.

After the regional taste testing, participants sampled the same taste stimuli described above with the whole mouth. Participants were asked to fill their mouths with ~ 10 mL of the taste solutions, swished for approximately 5 s (without swallowing) and then expectorate into a sink. After completing their ratings in the gLMS, participants rinsed twice with deionized water and waited 30 s before tasting the next stimuli.

#### Sucrose preferences

We assessed sucrose preferences by using the Monell forced-choice, paired comparison tracking technique, which is the NIH Toolbox gustatory measure recommended for young children [28]. Participants were presented with pairs of solutions that differed in sucrose concentration (from 3 to 36% g/v) and preferences were determined as previously described [28, 31].

### Statistical analyses

Separate one-way ANOVAs with group (Wolfram, T1D, and HC) as the between-subjects factor were used to determine whether groups differed in their smell sensitivity (n-butanol thresholds) and their ability to identify odorants (UPSIT and CCSIT scores). Fisher Exact Tests were conducted to detect differences in the frequency of abnormal smell sensitivity (i.e. dilution level for n-butanol detection thresholds or UPSIT scores below 10% of normative data specific for sex and age group). To examine taste sensitivity, separate two-way mixed ANOVAs were conducted for each taste stimuli (sucrose, sodium chloride and quinine hydrochloride) and for each region (tip of the tongue and whole mouth). The mixed ANOVAS included group (Wolfram, T1D, and HC) as the between-subjects factor and the three concentrations of each taste stimuli as the within-subject factor. When ANOVAs revealed significant differences, post hoc Fisher least significant difference analyses were conducted. The criterion for significance in all analyses was set at α = 0.05. All analyses were performed in Statistica v.13.3.

## Results

### Olfactory sensitivity

**Fig. 1:**
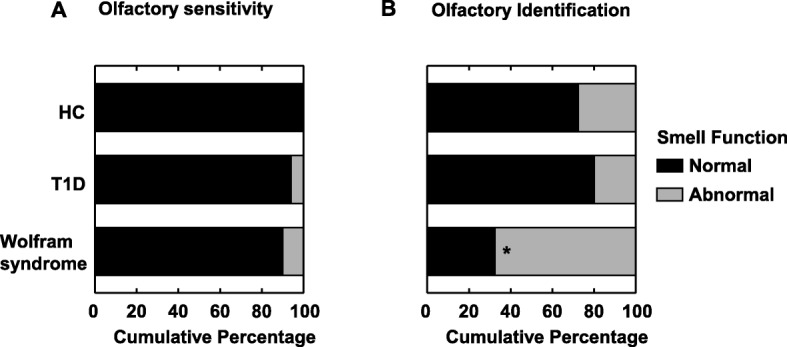
Olfactory function in participants with Wolfram syndrome and in two control groups: healthy controls (HC) and participants with Type 1 Diabetes Mellitus (T1D). **a** Olfactory sensitivity: Cumulative percentage of participants with a normal (in black) or abnormal (in grey) n-butanol detection thresholds (i.e. below 10% of normative data specific for sex and age group). **b** Olfactory identification: Cumulative percentage of participants with a normal (in black) or abnormal (in grey) UPSIT scores (i.e. below 10% of normative scores specific for sex and age group). *P < 0.05 compared to the two control groups

There were no significant differences between groups for n-butanol detection thresholds (Mean ± SEM; HC: 8.4 ± 0.8; T1D: 7.6 ± 0.8 and Wolfram syndrome: 7.3 ± 0.4; F _(2, 72)_ = 0.82, *P* = 0.44) or the percentage of participants in each group whose detection threshold was below the 10th percentile for age and sex matched normative data (*P* > 0.49; Fig. 1). Detection thresholds for one participant with Wolfram syndrome and for 18 participants in the control groups (7 in T1D and 11 in HC) were not available due to participants having a stuffy nose the day of testing or due to technical issues.

### Olfactory identification

The Wolfram group had lower UPSIT scores (F _(2, 91)_ = 9.97, *P* < 0.001) and CC-SIT scores (F _(2, 90)_ = 3.3, *P* < 0.05) than the HC and T1D groups (Mean ± SEM, UPSIT scores: HC: 30.1 ± 1.0; T1D: 31.6 ± 1.1 and Wolfram syndrome: 25.1 ± 1.2; CC-SIT scores: HC: 8.6 ± 0.5; T1D: 8.9 ± 0.5 and Wolfram syndrome: 7.3 ± 0.4). Participants with Wolfram syndrome were more likely to have an UPSIT score ≤ 10th percentile for age and sex matched normative data, than those in the HC and T1D groups (*P* < 0.0001; Fig. 1). While performance in the UPSIT positively correlated with age in both HC (*r* = 0.41; *P* < 0.05) and T1D groups (r = 0.62; *P* < 0.005)), there was no relationship with age in the Wolfram syndrome group (*r* = − 0.03; *P* > 0.85), suggesting early appearing deficits in smell identification.

### Taste intensity

Because preliminary data analysis for taste intensity showed no significant differences between the T1D and HC control groups, the two control groups were combined and henceforth referred to as “Combined Control Group”. Six participants in the Combined Control Group did not complete the test with quinine and therefore the final sample for both tip of the tongue and whole mouth procedure for bitter intensity is 25.

### Tip of the tongue

**Fig. 2:**
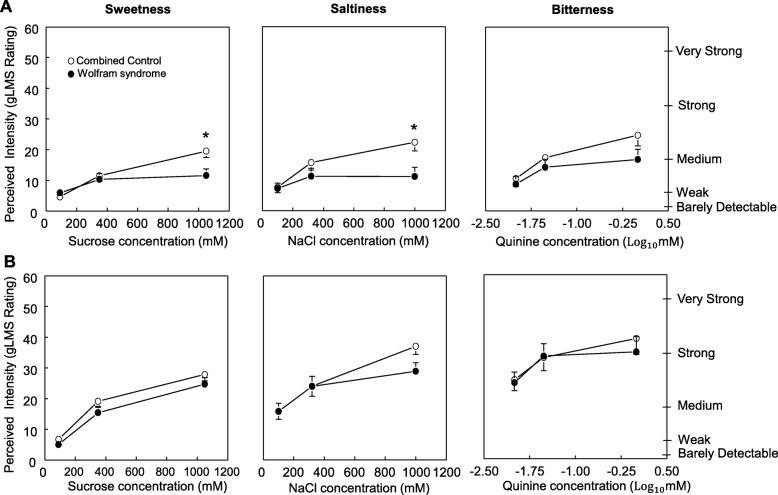
Taste function in participants with Wolfram syndrome and in the average of the two control groups without Wolfram syndrome (Combined controls). Perceived sweetness of increasing concentrations of sucrose, saltiness of increasing concentrations of sodium chloride, and bitterness of increasing concentrations of quinine hydrochloride. **a** Taste perception in the tip of the tongue and **b** in the whole mouth. The right axis shows descriptors visualized by participants when using the general labeled magnitude scale (gLMS). The left axis shows numbers corresponding to those descriptors on the scale. These numbers are not seen by subjects, but experimenters receive them from the computer program. Data are mean values ± SEM. *P < 0.05 compared to Combined control group

The Wolfram group perceived less sweetness in the highest sucrose concentration and less saltiness in the highest NaCl concentration than the Combined control group (Group x Concentration for sucrose: F _(2,114)_ = 4.42; *P* = 0.014; for NaCl: F _(2,114)_ = 4.44; *P* = 0.014; Fig. 2a). However, there were no significant differences between groups in bitterness perception for quinine (*P* > 0.16).

### Whole mouth

Taste intensity increased progressively and similarly between groups with an increase in stimulus concentration (sucrose sweetness: *P* < 0.00001, NaCl saltiness: *P* < 0.00001 and quinine bitterness: *P* < 0.00001). There were no interactions between groups and concentration for any of the assessed taste stimuli (all *P* values > 0.39; Fig. 2b).

### Sucrose preference

There were no differences in the preferred sucrose concentration between Wolfram and the Combined Control groups (F _(1, 85)_ = 0.13; *P* = 0.72; mean sucrose preferred by Wolfram = 18.6 ± 1.6 and Combined Control = 17.8 ± 1.4).

## Discussion

There are three main findings of our cross-sectional study. First, the current results confirm that Wolfram syndrome is associated with olfactory dysfunction [20]. Second, they further clarify that such olfactory impairment is qualitative (i.e. decreased ability for smell identification) and not due to olfactory insensitivity or secondary to having insulin-dependent diabetes. Third, our findings also suggest that, in contrast to the sense of smell (and vision and audition); the sense of taste is overall well conserved in individuals with Wolfram syndrome.

Experiences with certain odors and their names can be culturally specific; therefore, it could be argued that the lower ability to identify odors in the Wolfram syndrome group is due to the fact that many of the participants in this group, unlike those in the control groups, were coming from other countries and other cities in the United States. However, results from the analysis that used a subset of UPSIT items, which comprise the validated Cross-Cultural Smell Identification Test (CC-SIT) were similar to results with the full UPSIT, suggesting the observed differences between groups in the odor identification test were likely associated to Wolfram syndrome and not due to culturally related differences.

The finding that Wolfram syndrome is more related to a qualitative than a quantitative olfactory dysfunction is consistent with findings in patients with neurological conditions and suggest a smell impairment of central origin. For example, patients with focal unilateral excision in the temporal lobe or orbitofrontal cortex [32] have a marked impairment in olfactory identification but normal smell detection thresholds. Notably, pointing to a critical role of the orbitofrontal cortical region in higher-order olfactory tasks, smell function in patients with frontal-lobe excision sparing the orbital cortex left, or with parietal or central area lesions was unaffected [32]. Because the orbitofrontal cortex receives indirect projections from temporal regions via the medial dorsal nucleus of the thalamus, Jones-Gotman and Zatorre, hypothesized that smell identification deficits observed after temporal-lobe brain injury might be due to the disruption of these projections to the orbitofrontal region [32]. Consistent with this hypothesis, data from preclinical models [33, 34] and patients with thalamic lesions [35] show impairment in olfactory identification with normal detection. Another brain region that is not traditionally considered part of the olfactory system but that has been associated with olfactory performance is the cerebellum [36, 37]. Data from a recent study in healthy adults showed an association between reduced cerebellar gray matter volume and reduced odor identification but not odor detection or discrimination [37]. Moreover, patients with unilateral cerebellar lesions also have impaired olfactory identification with normal detection thresholds [36]. Interestingly, we have found that, compared with age and sex equivalent controls, patients with Wolfram syndrome had decreased volume in thalamus and cerebellar cortex [11].

Although our results strongly suggest a smell impairment of central origin, an alternative mechanism, although not mutually exclusive, is that, as shown for aging, a loss of specificity to olfactory stimuli in the peripheral olfactory system could also contribute to a decline in odor identification [38]. Specifically, in-vitro studies of biopsies of human olfactory sensory neurons revealed that unlike cells from younger donors, which were highly selective in the odors to which they responded, cells from older donors responded to multiple odor stimuli (i.e. were more “broadly tuned”). Future studies in Wolfram patients should assess olfactory discrimination in addition to olfactory identification to better advance our understanding of the olfactory dysfunction observed in this group.

In contrast to the deficits in smell identification, Wolfram patients have mostly intact taste. We observed a blunted response to taste stimuli in the tip of the tongue, but normal taste function when assessed in the whole mouth in Wolfram syndrome. The resilience of gustation in Wolfram syndrome, in comparison to dysfunction in the other senses, is likely due to the remarkable redundancy of the taste system: our most guarded sensory system [39]. Unlike other sensory modalities, which rely on one cranial nerve, taste signals are transmitted from the taste buds to the brain via three cranial nerves. In addition, not only are taste receptor cells continuously being replaced in the taste buds (every 9 to 15 days), but entire taste buds can be removed and they will fully regenerate [40]. The population with Wolfram syndrome evaluated in this study is relatively young and possibly has just started to develop a localized taste dysfunction specifically in the tip of the tongue, which is innervated by a branch of the facial nerve that includes the chorda tympani [41]. However, because there is a central mutual inhibition between the cranial nerves, when signals from the tip of the tongue are blunted, signals from the other regions of the tongue are intensified such that the net result is normal whole-mouth taste perception [41].

Limitations of the study include its cross-sectional design and possible participation bias. Wolfram patients were recruited who were relatively early in the disease process and could attend the clinic in St. Louis. Thus, participants who were more severely affected by the disease may have been excluded. Another limitation of the study is that due to time availability, olfactory discrimination, a third component that could shed some light on olfactory dysfunction etiology, was not assessed. Longitudinal studies of these participants are needed to better understand olfactory and taste function with disease progression.

## Conclusions

Using an extensive battery of well-validated psychometric tests, we examined smell and taste perception in a relatively young sample of patients with Wolfram syndrome and in control groups. Wolfram was associated with qualitative olfactory dysfunction that was not secondary to olfactory insensitivity or diabetes. In contrast, taste function was overall well-conserved, with the only exception of a regional decreased perception of taste intensity in the anterior tongue. Future longitudinal studies of taste and smell perception in patients with Wolfram syndrome will be important to determine the potential use of the chemical senses as clinical markers of disease progression.

## Data Availability

The raw datasets supporting the conclusion of this article are not publicly available because given that the sample size of our Wolfram syndrome patient group is relatively small and the disease is rare, human participant characteristics such as sex and age could result in identification of individuals even after de-identification of the data. However, datasets are available from the corresponding author on reasonable request.

## Abbreviations

HC: Healthy Controls
NaCl: Sodium Chloride
T1D: Type 1 Diabetes
UPSIT: University of Pennsylvania smell identification test
WFS: Wolfram Syndrome

## References

[1] Barrett TG, Bundey SE, Macleod AF (1995). Neurodegeneration and diabetes: UK nationwide study of Wolfram (DIDMOAD) syndrome. Lancet (London, England).

[2] Inoue H, Tanizawa Y, Wasson J, Behn P, Kalidas K, Bernal-Mizrachi E (1998). A gene encoding a transmembrane protein is mutated in patients with diabetes mellitus and optic atrophy (Wolfram syndrome). Nat Genet.

[3] Amr S, Heisey C, Zhang M, Xia X-J, Shows KH, Ajlouni K (2007). A homozygous mutation in a novel zinc-finger protein, ERIS, is responsible for Wolfram syndrome 2. Am J Hum Genet.

[4] Fonseca SG, Ishigaki S, Oslowski CM, Lu S, Lipson KL, Ghosh R (2010). Wolfram syndrome 1 gene negatively regulates ER stress signaling in rodent and human cells. J Clin Investig.

[5] Ariyasu D, Yoshida H, Hasegawa Y (2017). Endoplasmic Reticulum (ER) Stress and Endocrine Disorders. Int J Mol Sci.

[6] Barrett TG, Bundey SE (1997). Wolfram (DIDMOAD) syndrome. J Med Genet.

[7] Barrett TG, Bundey SE, Fielder AR, Good PA (1997). Optic atrophy in Wolfram (DIDMOAD) syndrome. Eye (London, England).

[8] Karzon R, Narayanan A, Chen L, Lieu JEC, Hershey T (2018). Longitudinal hearing loss in Wolfram syndrome. Orphanet J Rare Dis.

[9] Eljamel S, Ghosh W, De Stone S, Griffiths A, Barrett T, Thompson R (2019). A cost of illness study evaluating the burden of Wolfram syndrome in the United Kingdom. Orphanet J Rare Dis.

[10] Hershey T, Lugar HM, Shimony JS, Rutlin J, Koller JM, Perantie DC (2012). Early brain vulnerability in Wolfram syndrome. PLoS One.

[11] Lugar HM, Koller JM, Rutlin J, Eisenstein SA, Neyman O, Narayanan A (2019). Evidence for altered neurodevelopment and neurodegeneration in Wolfram syndrome using longitudinal morphometry. Sci Rep.

[12] Mesholam RI, Moberg PJ, Mahr RN, Doty RL (1998). Olfaction in neurodegenerative disease - A meta-analysis of olfactory functioning in Alzheimer’s and Parkinson’s diseases.

[13] Jung HJ, Shin I-S, Lee J-E (2019). Olfactory function in mild cognitive impairment and Alzheimer's disease: a meta-analysis. Laryngoscope.

[14] Doty RL (2012). Olfaction in Parkinson's disease and related disorders. Neurobiol Dis.

[15] Morley JF, Cohen A, Silveira-Moriyama L, Lees AJ, Williams DR, Katzenschlager R (2018). Optimizing olfactory testing for the diagnosis of Parkinson’s disease: item analysis of the university of Pennsylvania smell identification test. NPJ Parkinson’s Disease.

[16] Waschbisch A, Volbers B, Struffert T, Hoyer J, Schwab S, Bardutzky J (2011). Primary diagnosis of Wolfram syndrome in an adult patient--case report and description of a novel pathogenic mutation. J Neurol Sci.

[17] Lessell S, Rosman NP (1977). Juvenile diabetes mellitus and optic atrophy. JAMA Neurol.

[18] Genís D, Dávalos A, Molins A, Ferrer I (1997). Wolfram syndrome: a neuropathological study. Acta Neuropathol.

[19] Jackson MJ, Bindoff LA, Weber K, Wilson JN, Turnbull DM, Ince P (1994). Biochemical and molecular studies of mitochondrial function in diabetes insipidus, diabetes mellitus, optic atrophy, and deafness. Diabetes Care.

[20] Marshall BA, Permutt MA, Paciorkowski AR, Hoekel J, Karzon R, Wasson J (2013). Phenotypic characteristics of early Wolfram syndrome. Orphanet J Rare Dis.

[21] Bischoff AN, Reiersen AM, Buttlaire A, Al-lozi A, Doty T, Marshall BA (2015). Selective cognitive and psychiatric manifestations in Wolfram syndrome. Orphanet J Rare Dis.

[22] Whitcroft KL, Cuevas M, Haehner A, Hummel T (2017). Patterns of olfactory impairment reflect underlying disease etiology. Laryngoscope.

[23] Hummel T, Sekinger B, Wolf SR, Pauli E, Kobal G (1997). ‘Sniffin’ sticks’: olfactory performance assessed by the combined testing of odor identification, odor discrimination and olfactory threshold. Chem Senses.

[24] Hummel T, Kobal G, Gudziol H, Mackay-Sim A (2007). Normative data for the “Sniffin’ Sticks” including tests of odor identification, odor discrimination, and olfactory thresholds: an upgrade based on a group of more than 3,000 subjects. Eur Arch Otorhinolaryngol.

[25] Doty RL, Shaman P, Dann M (1984). Development of the university of Pennsylvania smell identification test: a standardized microencapsulated test of olfactory function. Physiol Behav.

[26] Doty RL (1995). The smell identification test™ Adnibistration manual Haddon Heights.

[27] Doty RL, Marcus A, Lee WW (1996). Development of the 12-item cross-cultural smell identification test (CC-SIT). Laryngoscope.

[28] Coldwell SE, Mennella JA, Duffy VB, Pelchat ML, Griffith JW, Smutzer G (2013). Gustation assessment using the NIH toolbox. Neurology.

[29] Bartoshuk LM, Duffy VB, Green BG, Hoffman HJ, Ko CW, Lucchina LA (2004). Valid across-group comparisons with labeled scales: the gLMS versus magnitude matching. Physiol Behav.

[30] Green BG, Dalton P, Cowart B, Shaffer G, Rankin K, Higgins J (1996). Evaluating the ‘labeled magnitude scale’ for measuring sensations of taste and smell. Chem Senses.

[31] Pepino MY, Mennella JA (2005). Sucrose-induced analgesia is related to sweet preferences in children but not adults. Pain.

[32] Jones-Gotman M, Zatorre RJ (1988). Olfactory identification deficits in patients with focal cerebral excision. Neuropsychologia..

[33] Eichenbaum H, Shedlack KJ, Eckmann KW (1980). Thalamocortical mechanisms in odor-guided behavior: I. effects of lesions of the mediodorsal thalamic nucleus and frontal cortex on olfactory discrimination in the rat. Brain Behav Evol.

[34] Slotnick BM, Kaneko N (1981). Role of mediodorsal thalamic nucleus in olfactory discrimination learning in rats. Science.

[35] Sela L, Sacher Y, Serfaty C, Yeshurun Y, Soroker N, Sobel N (2009). Spared and impaired olfactory abilities after thalamic lesions. J Neurosci.

[36] Mainland JD, Johnson BN, Khan R, Ivry RB, Sobel N (2005). Olfactory impairments in patients with unilateral cerebellar lesions are selective to inputs from the Contralesional nostril. J Neurosci.

[37] Wabnegger A, Schienle A (2019). Cerebellar gray matter and olfactory performance. Chem Senses.

[38] Rawson NE, Cowart BJ, Kriete A, Pribitkin E, Gomez G, Restrepo D (2012). Age-associated loss of selectivity in human olfactory sensory neurons. Neurobiol Aging.

[39] Breslin PA (2013). An evolutionary perspective on food and human taste. Curr Biol.

[40] Spielman AI, Pepino MY, Feldman R, Brand JG (2010). Technique to collect fungiform (taste) papillae from human tongue. J Vis Exp.

[41] Kveton JF, Bartoshuk LM (1994). The effect of unilateral chorda tympani damage on taste. Laryngoscope.

